# Polydatin attenuates Alzheimer’s disease induced by aluminum chloride in rats: evidence for its antioxidant and anti-inflammatory effects

**DOI:** 10.3389/fphar.2025.1574323

**Published:** 2025-04-17

**Authors:** Seyede Nazanin Zarneshan, Sajad Fakhri, Amir Kiani, Fatemeh Abbaszadeh, Seyede Zahra Hosseini, Ehsan Mohammadi-Noori, Javier Echeverría

**Affiliations:** ^1^ Student Research Committee, Kermanshah University of Medical Sciences, Kermanshah, Iran; ^2^ Pharmaceutical Sciences Research Center, Health Institute, Kermanshah University of Medical Sciences, Kermanshah, Iran; ^3^ Regenerative Medicine Research Center, Health Technology Institute, Kermanshah University of Medical Sciences, Kermanshah, Iran; ^4^ Neurobiology Research Center, Institute of Neuroscience and Cognition, Shahid Beheshti University of Medical Sciences, Tehran, Iran; ^5^ Departamento de Ciencias del Ambiente, Facultad de Química y Biología, Universidad de Santiago de Chile, Santiago, Chile

**Keywords:** Alzheimer’s disease, aluminum chloride, polydatin, inflammation, matrix metalloproteinase, oxidative stress, catalase, glutathione

## Abstract

**Background:**

Considering the complex pathophysiological mechanisms behind Alzheimer’s disease (AD), a few drugs for managing related cognitive symptoms have been approved. The phytochemical resveratrol has shown promising anti-inflammatory and antioxidant effects in AD, but it has low bioavailability. Chemical modification of resveratrol to its glycosylated form, polydatin (PD), significantly increases its bioavailability and bioactivity.

**Purpose:**

The study aimed to investigate the therapeutic potential and mechanisms of action of PD against AD in rats.

**Material and method:**

AD was caused by an intraperitoneal (i.p.) administration of aluminum chloride (AlCl_3_). Six groups of six rats each were defined as sham, negative control (AlCl_3_), positive control (Donepezil), and treatments (PD 5, 10, and 20 mg/kg, i.p.). On days 7, 8, 14, and 15, the rats’ behavioral changes were assessed by the open field, Y-maze test, passive avoidance test, and elevated plus maze tests. At the end of the study, the blood samples were collected to assess the levels of glutathione (GSH), catalase (CAT), and nitrite, as well as the activity of matrix metalloproteinases (MMPs). Furthermore, hippocampal brain tissue was removed and used for histological investigations.

**Results and discussion:**

The findings revealed that PD injections at three different doses (5, 10, and 20 mg/kg) improved cognitive and other behavioral impairments. Furthermore, PD improved the antioxidant capacity by increasing GSH and CAT while decreasing serum nitrite levels. PD showed anti-inflammatory effects by reducing the activity of inflammatory MMP-9, while elevating the activity of anti-inflammatory MMP-2. PD also modulated pathogenic changes in the hippocampal brain tissue.

**Conclusion:**

PD alleviated cognitive and other behavioral impairments in AD rats by enhancing antioxidant defenses and reducing neuroinflammation.

## 1 Introduction

Dementia is a clinical situation defined as gradual dysfunction in memory, behavior, language, cognitive/visuospatial function, and personality, leading to the impairment of the ability to perform daily tasks (Crous-Bou et al., 2017). As the most prevalent dementia reason, Alzheimer’s disease (AD) has been a critically costly, burdensome, and fatal disease of the 21st century (Scheltens et al., 2021). AD is a very sophisticated and advanced neurodegenerative condition with multiple pathophysiological mechanisms (Querfurth and LaFerla, 2010). Amyloid beta (Aβ) and amyloid precursor protein (APP) are transmembrane proteins generally found in synapses. They are cleaved by α-secretase, followed by γ-secretase, and processed and removed appropriately in healthy non-amyloidogenic situations. Tau acts as a microtubule-stabilizing protein in neuronal axons in the healthy condition (Eratne et al., 2018). Extracellular Aβ plaque aggregates and intracellular neurofibrillary tangles (NFTs), which are made of hyperphosphorylated tau linked with microtubules, are thought to be the histological features of AD (Goedert, 2015). Aβ pathology is in a near interconnection with the inflammatory hypothesis in AD. Matrix metalloproteinases (MMPs) are among the proteins receiving increased attention for their function in neuroinflammation and neurodegeneration. MMP-2 and MMP-9 break Aβ monomers and oligomers, causing neurotoxicity (Iranpanah et al., 2024). AD has been related to iron accumulation and brain iron dysregulation, which interact with tau and Aβ pathologies, resulting in oxidative damage and reactive oxygen species, promoting neurodegeneration. Free radical buildup leads to synaptic dysfunction, inflammation, mitochondrial damage, oxidative stress, neurotransmitter deficits (particularly cholinergic), microglial activation, and, finally, neuronal death (Eratne et al., 2018). Critical neuroprotective mechanisms of active compounds have also been shown to be connected with antioxidant defense mechanisms (Ishak et al., 2024). Regulation of oxidative/antioxidative mediators (e.g., nitric oxide synthase) is also correlated with protective effects against brain and peripheral damage (Selamoglu-Talas et al., 2015; Sureda et al., 2023).

Substantial studies have focused on the possible involvement of aluminum (Al) in the AD process, owing to its recognized numerous and powerful neurotoxic effects. Al is the most common natural neurotoxic metal to which we are exposed (Bhattacharjee et al., 2014). As a result, identifying pharmaceuticals or natural compounds that protect against Al-mediated neuronal cell death is a potent method for neurodegenerative disorder prevention and treatment (Anwar et al., 2021).

The amount of fatalities from AD has climbed dramatically during the last 20 years, yet a cure is still unachievable. FDA-approved therapies for AD have not demonstrated a curative impact, but generally neither do they slow the pace of disease progression (Singh et al., 2024). For patients with AD dementia, cholinesterase inhibitors (e.g., physostigmine) and *N*-methyl-D-aspartate (NMDA) antagonists (e.g., memantine) are approved drugs (Mossello and Ballini, 2012). The currently authorized medications operate on a single target, however, importance is being given to numerous therapeutic techniques for designing and developing treatments that can impact several targets (Athar et al., 2021). As a result, there is a growing demand for innovative multi-target medicines. Numerous natural chemicals derived from edible and medicinal plants have been studied for possible pharmacological use. Natural substances are high in polyphenolic substances such as stilbenoids with antioxidant potentials (Fakhri et al., 2021a). Natural stilbenes have received far less attention (Freyssin et al., 2020). The antioxidant capacity of stilbene polyphenols has been linked with neuroprotective mechanisms (Sureda et al., 2023). However, several stilbenes polyphenols have been characterized solely in terms of their *in vitro* actions. In contrast, resveratrol has been extensively studied for its benefits both *in vitro* and *in vivo*, in murine and rat models, and even in clinical trials of AD (Witte et al., 2014; Moussa et al., 2017; Cicero et al., 2019; Ma et al., 2019; Broderick et al., 2020). However, because resveratrol undergoes substantial hepatic metabolism and is quickly eliminated by the kidneys, its bioavailability is limited (Gambini et al., 2015). Due to resveratrol’s limited bioavailability, newer molecules like its homolog (3,4′,5-trihydroxystilbene-3-β-D-glucoside, commonly called polydatin (PD), should be investigated. PD, is a glucoside derivative of resveratrol. The substitution of a hydroxyl group for a glucose unit distinguishes it from resveratrol (Tang, 2021). PD’s putative protein targets have been discovered. PD can penetrate the blood-brain barrier (BBB), which is critical in the treatment of brain disorders (Pan et al., 2018; Tang and Tan, 2019). In comparison to its counterpart, resveratrol, research on PD’s preventive function against AD is limited (Tang, 2021). We also previously reported the neuroprotective effects of PD amphiphilic chitosan nanocarriers in a rat model of AD (Zarneshan et al., 2025).

This research aims to investigate the potential mechanisms of action of PD against AD in an aluminum chloride-induced AD rat model.

## 2 Materials and methods

### 2.1 Animals

Male Albino Wistar rats (weighing 220–250 g) were obtained from the central animal house at Kermanshah University of Medical Sciences (KUMS). They were kept in an environment with an *ad libitum* supply of food and water, a standard light/dark cycle (i.e., 12 h/12 h), a relative temperature of 24°C ± 2°C, and a proper humidity (i.e., 60% ± 5%). All the experimental protocols were done based on the guidelines of the institutional animal care and ethics of KUMS (IR.KUMS.AEC.1401.050).

For 14 days, thirty-six rats were split up into six groups of six rats each. The sample size was calculated using the modified resource equation method (Arifin and Zahiruddin, 2017). The sham group just received intraperitoneal (i.p.) saline. The negative control group received AlCl_3_ (100 mg/kg, i.p.) and i.p. saline. The positive control group received donepezil (10 mg/kg, i.p.) after AlCl_3_ (100 mg/kg, i.p.). Groups IV, V, and VI received AlCl_3_ (100 mg/kg, i.p.) followed by three doses of PD (i.e., 5, 10, and 15 mg/kg, i.p.). The animals’ behavioral analyses were all done during the study timeline. The rats were subsequently sacrificed, and blood samples were used to measure serum levels of nitrite, glutathione (GSH), and catalase (CAT). In addition, the activity of MMP-2 and MMP-9 was measured, and hippocampal brain tissue was extracted and utilized for histopathological studies. The rats were assigned to groups using randomization and blinding in animal assignments, behavioral assessments, and biochemical analyses (Zarneshan et al., 2025).

### 2.2 Chemicals

Polydatin, donepezil, ammonium molybdate, aluminum chloride (AlCl_3_), sodium nitrite, *N*-(1-naphthyl) ethylenediamine dihydrochloride (NED), sodium dodecyl sulfate (SDS), 5,5′-dithiobis-(2-nitrobenzoic acid) (DTNB), coomassie brilliant blue G-250, acrylamide, and Triton X-100 were purchased from Sigma-Aldrich (St. Louis, MO).

### 2.3 Behavioral studies

#### 2.3.1 Open field test

The open field test is a common laboratory method designed to evaluate general locomotion and anxiety-related behavior (Lu et al., 2019). Depending on the animal being tested, it consists of a wall enclosed (high sufficient to prevent escape), circular, square, or rectangular, unknown space that is large enough to provide the impression of openness. It is possible to assess a range of activities, such as the number of squares that the rats traversed, rearing, and grooming (Vuralli et al., 2019).

#### 2.3.2 Passive avoidance test

According to the association formed between a particular unpleasant event and a certain environmental setting, the passive avoidance task is a fear-motivated test used to evaluate fear-based conditioned avoidance learning and memory/behavior in rats (Giménez De Béjar et al., 2017). In this project, normal animals come to the recognition that staying away from a specific location will help them prevent an unpleasant event. This test is carried out in two compartments of similar size, one of which is bright (white and lit by a 24 V-10 W bulb), and the other of which is dark (black and dark), both of which are divided by a gate. The acquisition (training) phase and the test phase were the two stages of testing. Throughout the initial training phase, animals were placed in the illuminated box and permitted a maximum of 5 min to pass the gate and enter the dark box. Animals accessing the dark box with the gate closed suffered a 2-s foot shock. Following the foot shock, each animal was allowed 20 s in the dark cage to acclimate (0.5 mA). After 20 s, the animals were taken away from the apparatus and placed back in their cages. On day two of the test phase, testing was conducted in the same manner as on day one, but without the use of a foot shock in the dark box (Wu et al., 2020).

#### 2.3.3 Y-maze test

The Y-maze is a standard method for testing spatial memory. Three arms diverge at a 120° angle from the device’s center. Throughout the test, the baffles of two randomly selected arms were opened. The start arm is preferred by the two, and the new arm is allocated to the arm with the baffle still attached (Shi et al., 2021). Beginning by assigning the letters A, B, and C to the maze’s three arms, then start recording. Next, consider the overall number of arm entries and alternations recorded. Finally, use the following formula to determine the percent (%) alternation: $\%\text{ Alternation }\left(\text{SAP}\right)=\left(\text{Number of Alternations}/\left[\text{Total number of arm entries}-2\right]\right)\times 100$ (Kraeuter et al., 2019).

#### 2.3.4 Elevated plus maze test

The elevated plus-maze is the most frequently employed task to study anxiety-like behavior overall, and in transgenic models of AD in particular (Pentkowski et al., 2021). The testing apparatus looks like a cross and has two arms. The arm enclosed by walls is known as the contained arm, and the arm without walls is known as the open arm (MacLellan et al., 2009). Rats were placed at the center of defined arms and allowed to explore for 5 min. Spent time in open arms, overall distance traveled, transfer latency, and initial transfer latency (ITL) were recorded (Zarneshan et al., 2025).

### 2.4 Biochemical tests

#### 2.4.1 Glutathione assay

GSH was determined using Ellman’s assay (Ellman, 1959). In this research, each well received 50 mL of phosphate buffer with a pH of 7.4 and 60 mL of rat serum sample. Following the addition of 100 mL of DTNB, a yellow complex was seen, which was then incubated for 10 min at 37°C before the absorbance at 412 nm was detected using an ELISA reader. Lastly, the percentage of optical absorption groups that differed from the reference group (healthy rats) was reported.

#### 2.4.2 Catalase assay

In this experiment, CAT changes using the Aebi method were evaluated by measuring hydrogen peroxide (Aebi, 1980). First 20 mL of this study’s serum samples from the rats in each group were added to each well, followed by the addition of 100 mL of hydrogen peroxide at a concentration of 65 mM, 4 min of incubation at 25°C, and the addition of 100 mL of ammonium molybdate at a concentration of 32.4 mM to terminate the reaction. Finally, using an ELISA reader, the yellow molybdate and hydrogen peroxide complex were quantified at 405 nm. Eventually, the control group’s optical absorption % was detected to be different from the other groups (Zarneshan et al., 2025).

#### 2.4.3 Nitrite assay

Nitrite was measured using the Griess method (Granger et al., 1995; 1996) through a deproteinization by adding zinc sulfate powder. First, sulfanilamide (in 5% HCl) and naphthyl ethylene diamine dihydrochloride (NEDD; 0.1% in distilled water) were prepared. In a 96-well plate, 50 μL of sulfanilamide solution was combined with 100 μL of the prepared serum samples and incubated in the dark for 5 min. After that, 50 μL of NEDD solution was added, and incubation was done at 37°C for 30 min. After that, the purple complex was formed, and its absorbance was measured at 540 nm using a plate reader. Lastly, the concentration of nitrite in the serum of rats was reported in mM (Zamani et al., 2025).

### 2.5 Zymography

Gelatin zymography was used to measure the gelatinolytic activity of MMPs. Polyacrylamide gels containing sodium dodecyl sulphate were copolymerized with 0.1% gelatin. Following the loading of blood samples (equal to 100 µg of total protein content as determined by the Bradford assay), electrophoresis was performed using a mini-gel slab device called the Mini Protean III (Bio-Rad, Hercules, CA) at a fixed voltage of 150 V. The gel was shaken for an hour while being washed in renaturation buffer with 2.5% Triton X-100 (in 50 mM Tris-HCl). Incubation buffer containing 10 mM CaCl_2_, 0.02% NaN_3_, and 0.15 M NaCl in 50 mM Tris-HCl was then used to incubate the gel for 18 h at 37°C. After incubation, the gels were stained for 40 min with Coomassie brilliant blue G-250 (0.5% in 10% acetic acid and 30% methanol) and then destained with a solution of distilled water, glacial acetic acid, and methanol for 60 min. ImageJ^®^ software (NIH) was used to analyze images and assess MMP-9 and MMP-2 activity by evaluating the intensity of clear bands against colored backgrounds (Fakhri et al., 2021b; Hashemi et al., 2024).

### 2.6 Histopathological assessments

All animals were given 100 mg/kg of ketamine and 10 mg/kg of xylazine for scarification after the behavioral tests. After being carefully removed, the hippocampal tissue was stored in 200 mL of 0.1 M PBS and 4% paraformaldehyde. Tissues were sliced to a 5 mm thickness, and Hematoxylin and Eosin (H&E) stain was then applied. The histological changes were examined under a light microscope, and data were gathered at ×400 magnification (Zarneshan et al., 2025).

### 2.7 Statistical analysis

Regarding analyzing the data, Prism 8.4.3 software (GraphPad Software Inc., San Diego, CA, USA) was employed. To evaluate the normality of the data distribution, the Shapiro-Wilk test was performed. One-way and two-way analyses of variance (ANOVA) were conducted, followed by Tukey or Bonferroni *post hoc* analysis. The data were presented as mean ± standard error of the mean (SEM), with a statistical significance of *P* < 0.05.

## 3 Results

### 3.1 Behavioral tests

#### 3.1.1 Polydatin effects on the open field test

**FIGURE 1:**
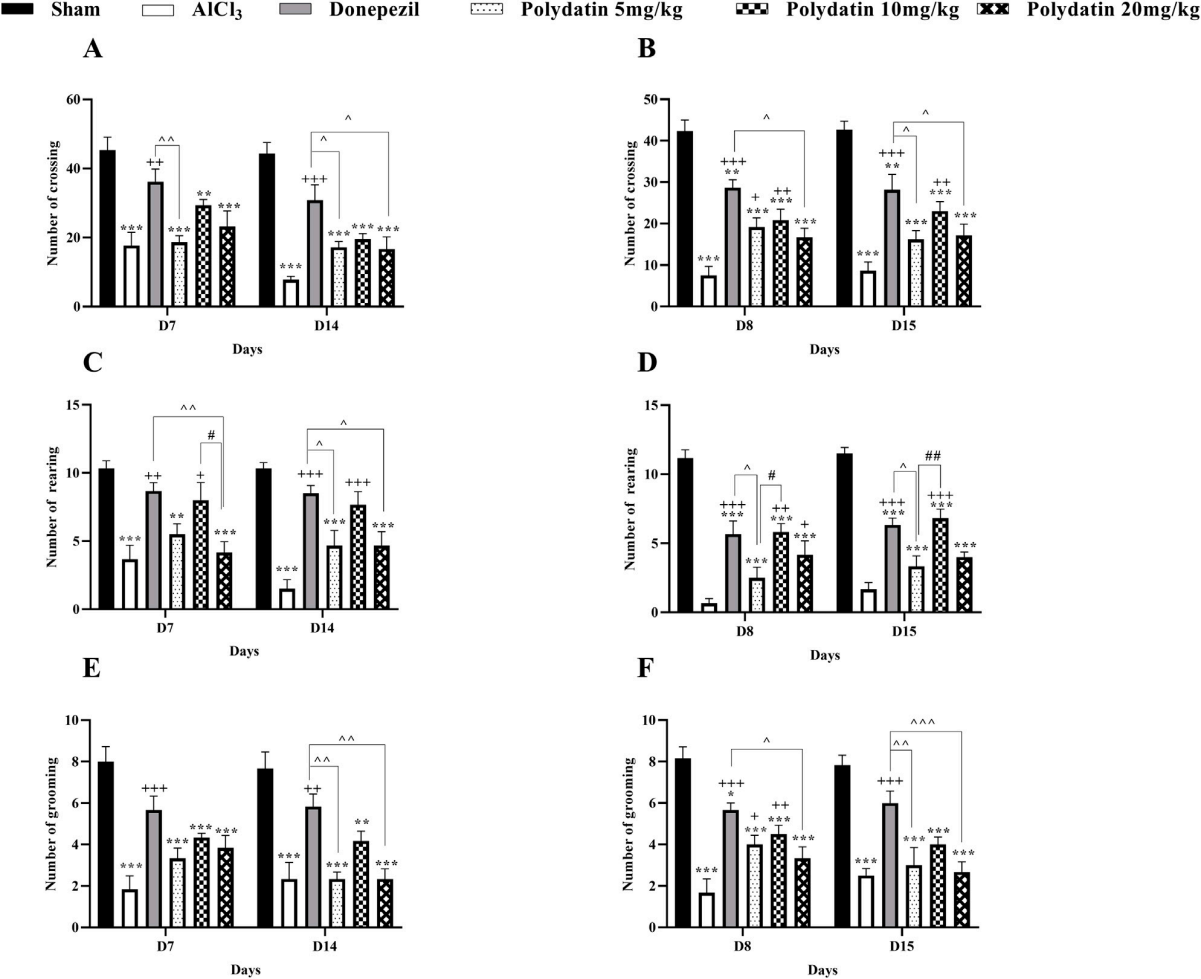
Polydatin attenuated anxiety-related behaviors in an AlCl_3_-induced rat model of AD. Number of **(A, B)** Crossing, **(C, D)** Rearing, and **(E, F)** Grooming. The data passed the Shapiro-Wilk test for normality. The two-way ANOVA with Bonferroni *post hoc* analysis was employed, and data were presented as mean ± SEM (*n* = 6). **P < 0.01, ***P < 0.001 vs. Sham group; ^+^ *P* < 0.05, ^++^ *P* < 0.01, ^+++^ *P* < 0.001 vs. AlCl_3_ group; ^^*P* < 0.05, ^^^^ *P* < 0.01, ^^^^^^ *P* < 0.001 vs. Donepezil group; ^#^ *P* < 0.05, ^##^ *P* < 0.01 vs. Polydatin 10 mg/kg groups.

Figure 1 represents the general locomotion and anxiety-related behaviors in a novel setting as indicated by the open field test. On every day of the open field test, a statistically significant (*P* < 0.001) reduction was noted in the quantity of crossing, rearing, and grooming in the AlCl_3_ group as compared to the control group. Treatment with PD in doses of 5, 10, and 20 mg/kg significantly increased the movement of rats compared with the AlCl_3_ group (*P* < 0.001). This increase in crossing in PD 10 mg/kg and donepezil is more significant (*P* < 0.001). Also, treatment with PD caused a noteworthy increase compared to the AlCl_3_ group in the number of rearing and grooming in rats (*P* < 0.01 and *P* < 0.05). Therefore, the use of PD improved movement (locomotion) and anxiety behavior caused by AlCl_3_.

#### 3.1.2 Polydatin effects on passive avoidance test

**FIGURE 2:**
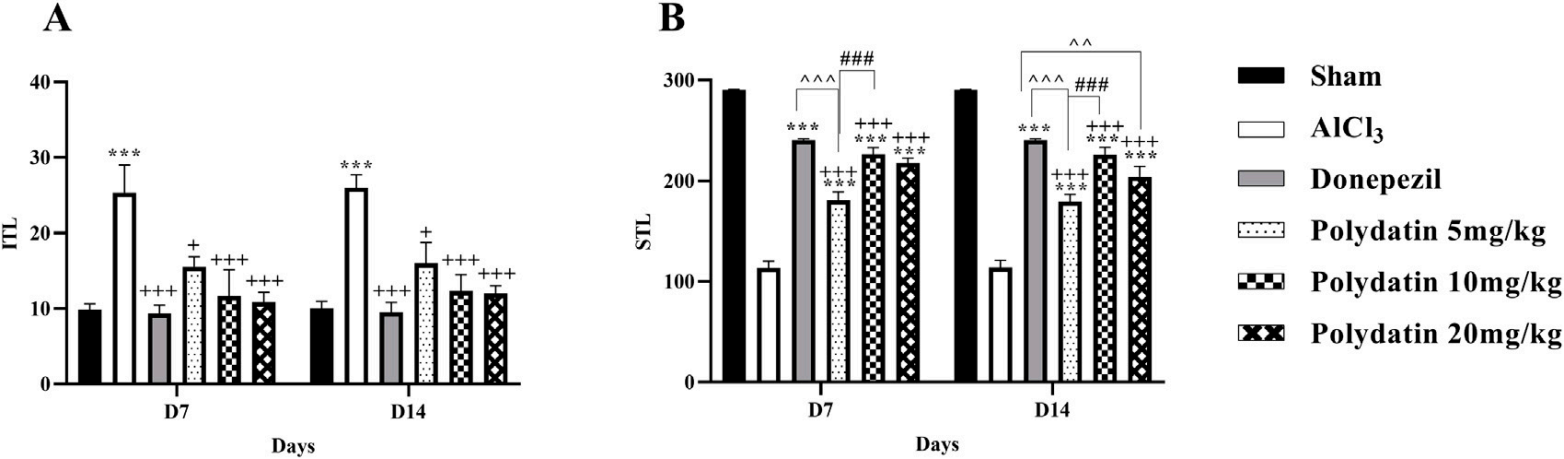
Polydatin regulated related behaviors in the passive avoidance test during an AlCl_3_-induced rat model of AD. **(A)** Initial transfer latency (ITL) and **(B)** Step-through latency (STL). The data passed the Shapiro-Wilk test for normality. The two-way ANOVA with Bonferroni *post hoc* analysis was employed, and data were presented as mean ± SEM (*n* = 6). ***P < 0.001 vs. Sham group; ^+^ *P* < 0.05, ^+++^ *P* < 0.001 vs. AlCl_3_ group; ^^^^ *P* < 0.01, ^^^^^^ *P* < 0.001 vs. Donepezil group; ^###^ *P* < 0.001 vs. Polydatin 10 mg/kg groups.

Figure 2 presents the results of the passive avoidance test for the avoidance learning, memory, and behavior of rats. The purpose of this test was to see if the animal could learn to react quickly to unpleasant stimuli like electric shocks. Because they are nocturnal creatures, rodents, like rats, prefer the dark. After training on the first day (learning), the rat is supposed to recall the negative stimulus in this test, which is an electric shock, and not go into the dark room the following day. The initial transfer latency time (ITL) on days 7 and 14, and step through latency time (STL) to enter the dark section were recorded on days 8 and 15. Considering the inherent tendency of rats to be in the dark part, the amount of ITL time in the AlCl_3_ group increased compared to the control group on days 7 and 14 (*P* < 0.001). While the consumption of PD in doses of 5, 10, and 20 mg/kg causes a noteworthy reduction in ITL time in comparison with the AlCl_3_ group (*P* < 0.05, *P* < 0.001, and *P* < 0.001). In the PD-receiving groups, the ITL time reduction in the group receiving 5 mg/kg PD is less than the other treatment groups (*P* < 0.05), and there was no considerable difference between the PD 10 and 20 groups and the group receiving donepezil. In the AlCl_3_ group compared to the control group, a noteworthy decrease in STL time was observed (*P* < 0.001), which indicates memory deterioration in the AlCl_3_ group. Treatment with PD in doses of 5, 10, and 20 mg/kg causes significant improvement and an increase in STL in comparison with the AlCl_3_ group (*P* < 0.001). Among treatment groups with PD, memory improvement in PD 10 mg/kg was more than in other groups. In addition, there was no major difference between groups receiving PD 10 mg/kg and the group receiving donepezil (*P* < 0.001).

#### 3.1.3 Polydatin effects on Y-maze test

**FIGURE 3:**
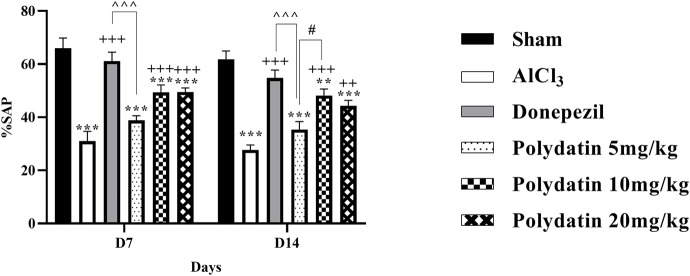
Polydatin modulated spatial memory in an AlCl_3_-induced rat model of AD. The data passed the Shapiro-Wilk test for normality. The two-way ANOVA with Bonferroni *post hoc* analysis was employed and data was presented as mean ± SEM (*n* = 6). **P < 0.01, ***P < 0.001 vs. Sham group; ^++^ *P* < 0.01, ^+++^ *P* < 0.001 vs. AlCl_3_ group; ^^^^^^ *P* < 0.001 vs. Donepezil group; ^#^ *P* < 0.05 vs. Polydatin 10 mg/kg groups.

Figure 3 presents the Y-maze results for the spatial memory of rats. The results of this test show an important defect in spatial memory and a decrease in general activity in the AlCl_3_ group compared to the control group, with a decrease in the percentage of non-repetitive intervals and the total number of entering the arm (SAP%) (*P* < 0.001). Also, treatment with PD in different doses increased the SAP% in comparison with the AlCl_3_ group, especially at the 10 mg/kg and 20 mg/kg dosage, which was significantly higher than that of the AlCl_3_ group (*P* < 0.001 and *P* < 0.01) and able to recover short-term memory and general activity in rats and averts memory loss caused by AlCl_3_. Additionally, there was no significant difference in %SAP between the NPD and donepezil groups (*P* < 0.001).

#### 3.1.4 Polydatin effects on the elevated plus maze test

**FIGURE 4:**
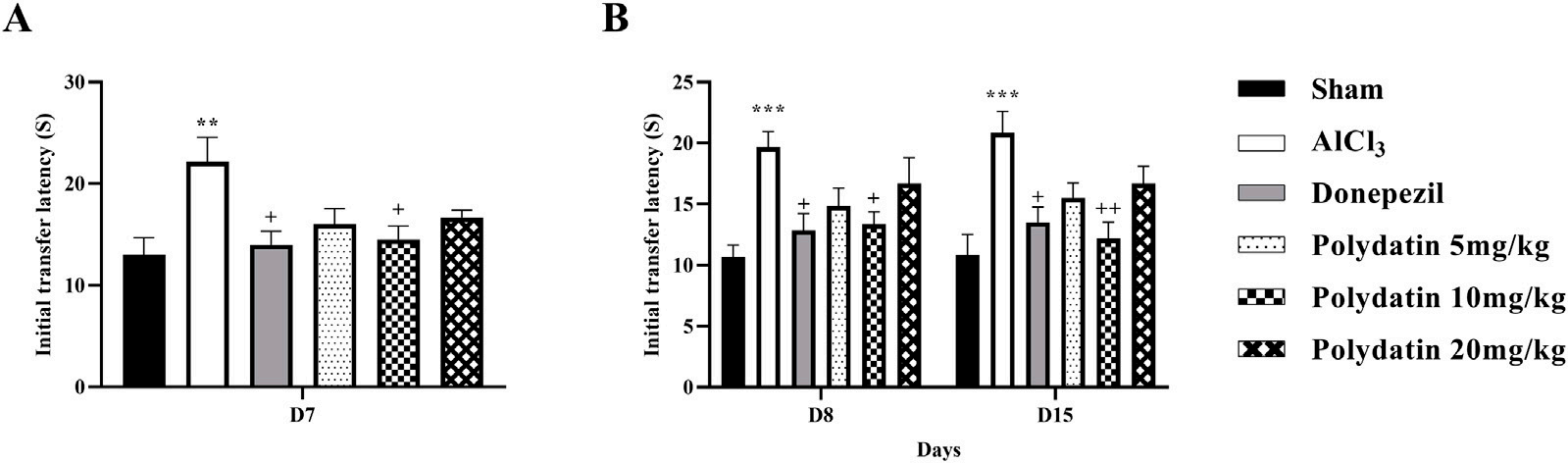
Polydatin effects on anxiety-like behavior and memory in an AlCl_3_-induced rat model of AD. **(A)** Initial transfer latency (ITL-D7) and **(B)** ITL (D8-D15). The data passed the Shapiro-Wilk test for normality. The one-way and two-way ANOVA with Tukey and Bonferroni *post hoc* analysis was employed, and data were presented as mean ± SEM (*n* = 6). **P < 0.01, ***P < 0.001 vs. Sham group; ^+^ *P* < 0.05, ^++^ *P* < 0.01 vs. AlCl_3_ group.

Figure 4 indicates how PD affects anxiety-like behavior, such as the transfer latency of rats given AlCl_3_ treatment in the elevated plus-maze. The purpose of this test was to measure the rats’ post-learning memory. In this test, the time required to enter the arm for each rat on day 7 (learning) as the ITL, and the time required to enter the arm on days 8 and 14 (memory) as the transfer latency time, was recorded. Reduction in transfer latency time after determining the ITL time indicates the improvement of the rats’ diagnostic memory. In this test, a significant difference was observed in the learning time between the group receiving AlCl_3_ and the control group (*P* < 0.01).

Similarly, in this test, the group receiving AlCl_3_ showed an increase in the transfer latency time of the first and second transfer (memory) on days 8 and 14, respectively, according to the time ITL on the 7th day, compared to the control group, treatment with PD showed a memory enhancement (*P* < 0.05). This effect is more visible at the dose of 10 mg/kg PD.

### 3.2 Biochemical tests

#### 3.2.1 Polydatin effects on catalase assay

**FIGURE 5:**
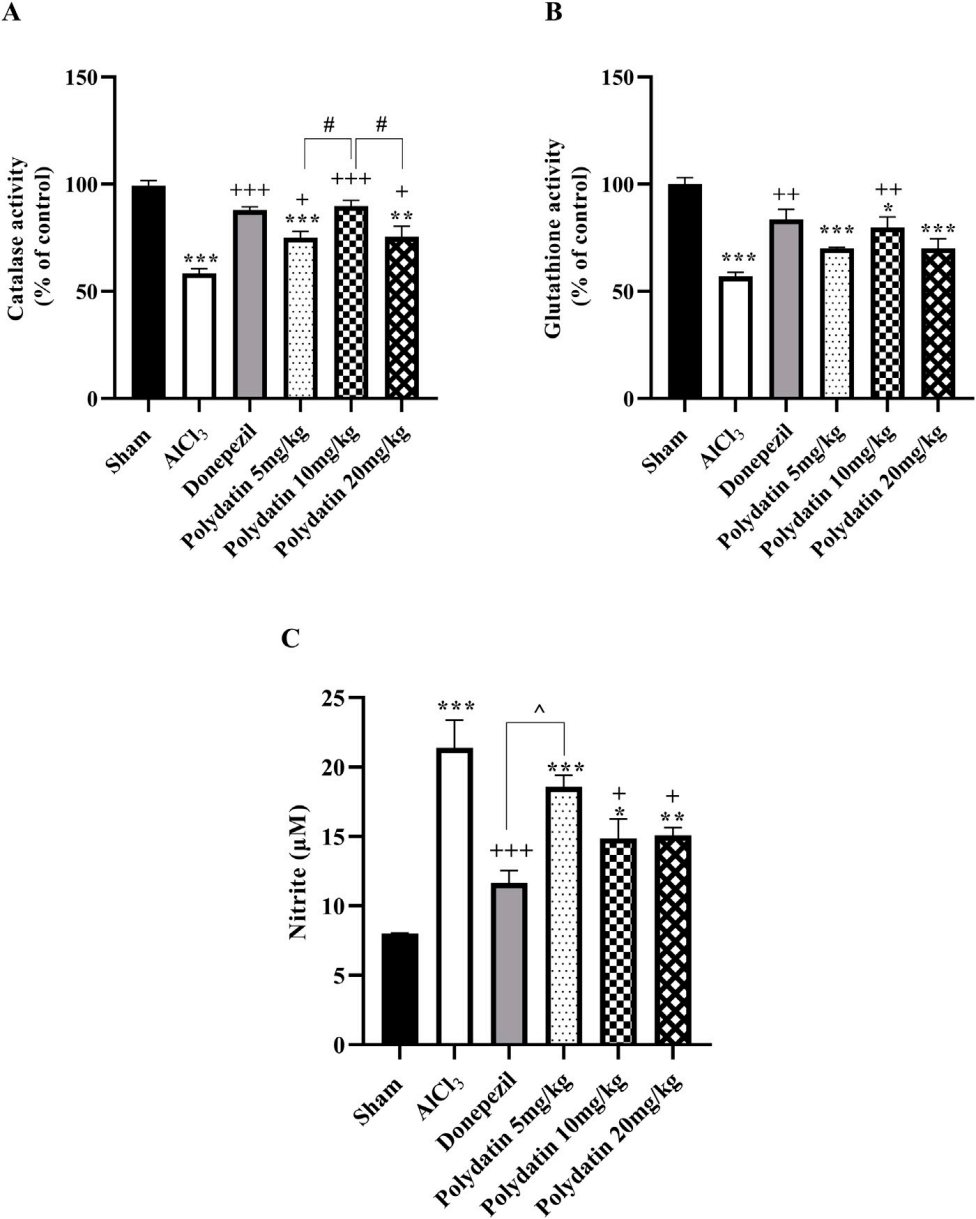
Polydatin regulatory roles on oxidative stress in a rat model of AD induced by AlCl_3_. **(A)** Catalase, **(B)** Glutathione, and **(C)** Nitrite. The data passed the Shapiro-Wilk test for normality. The one-way ANOVA with Tukey *post hoc* analysis was employed, and data were presented as mean ± SEM (*n* = 6). *P < 0.05, **P < 0.01, ***P < 0.001 vs. Sham group; ^+^ *P* < 0.05, ^++^ *P* < 0.01, ^+++^ *P* < 0.001 vs. AlCl_3_ group; ^^ *P* < 0.05, ^^^^ *P* < 0.01 vs. Donepezil group; ^#^ *P* < 0.05 vs. Polydatin 10 mg/kg groups.

The amount of serum CAT was measured as an antioxidative stress factor, and it is desirable not to decrease it. The results in Figure 5A reveal that consuming AlCl_3_ induces an enormous decrease in serum CAT levels (*P* < 0.001); whereas the PD groups have a considerably lower difference in serum CAT levels than the sham group (*P* < 0.01 and *P* < 0.001). CAT quantity improvement in PD 10 mg/kg was more than in other PD groups (*P* < 0.05), and there was no statistically significant distinction between rats receiving PD 10 mg/kg and those receiving donepezil.

#### 3.2.2 Polydatin effects on glutathione assay

Serum GSH has been investigated as an antioxidative stress component and should not be decreased. The results in Figure 5B show that the consumption of AlCl_3_ causes a major decrease in the serum GSH level (*P* < 0.01), while the treatment groups showed a significant difference in the serum GSH level compared to the sham group exclusively 10 mg/kg, thus causing neuroprotection by inhibiting oxidative stress (*P* < 0.05).

#### 3.2.3 Polydatin effects on nitrite assay

The level of serum nitrite has been identified as an oxidative stress factor and should not be increased. The results in Figure 5C demonstrate that intake of AlCl_3_ resulted in an important reduction in serum nitrite level compared to the sham group (*P* < 0.001), whereas consumption of PD resulted in a substantial decrease in NO level compared to the group (*P* < 0.001). As a result, the usage of PD could result in neuroprotection by decreasing oxidative stress, particularly in a dose of 10 mg/kg (*P* < 0.001). Crucially, there were no discernible variations in these parameters between the donepezil and PD treatment groups (*P* < 0.01).

### 3.3 Polydatin effects on MMP-2 and MMP-9

**FIGURE 6:**
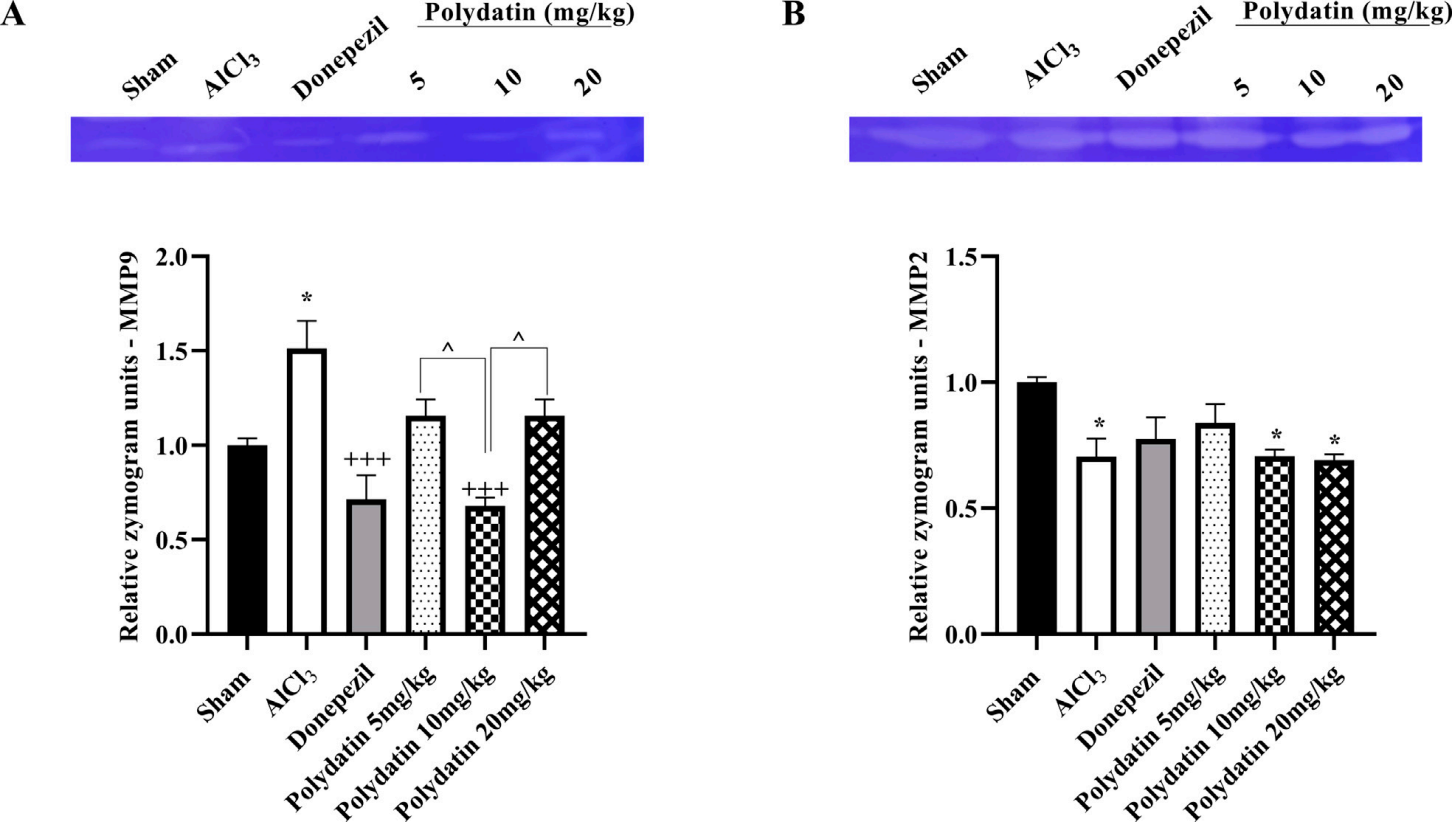
Effects of polydatin on MMPs levels in a rat model of AD induced by AlCl_3_. **(A)** MMP-9 and **(B)** MMP-2. The data passed the Shapiro-Wilk test for normality. The one-way ANOVA with Tukey *post hoc* analysis was employed, and data were presented as mean ± SEM. *P < 0.05 vs. Sham group; ^+++^ *P* < 0.001 vs. AlCl_3_ group; ^^ *P* < 0.05 vs. Donepezil group.

The assessment of MMP gelatinase activity across different groups indicated that MMP-9 activity was significantly elevated in the AlCl_3_ group compared to the sham group (*P* < 0.05, Figure 6A). Treatment with PD effectively reduced MMP-9 gelatinase activity (*P* < 0.001). Interestingly, the 10 mg dose of PD not only decreased MMP-9 activity but also showed effects comparable to those of donepezil (positive control). Moreover, the AlCl_3_ group demonstrated markedly lower MMP-2 levels than the sham group (*P* < 0.05, Figure 6B). Conversely, the 5 mg/kg PD-treated group exhibited a partial increase in MMP-2 levels when compared to the AlCl_3_ group.

### 3.4 Polydatin effects on histopathological assessments

**FIGURE 7:**
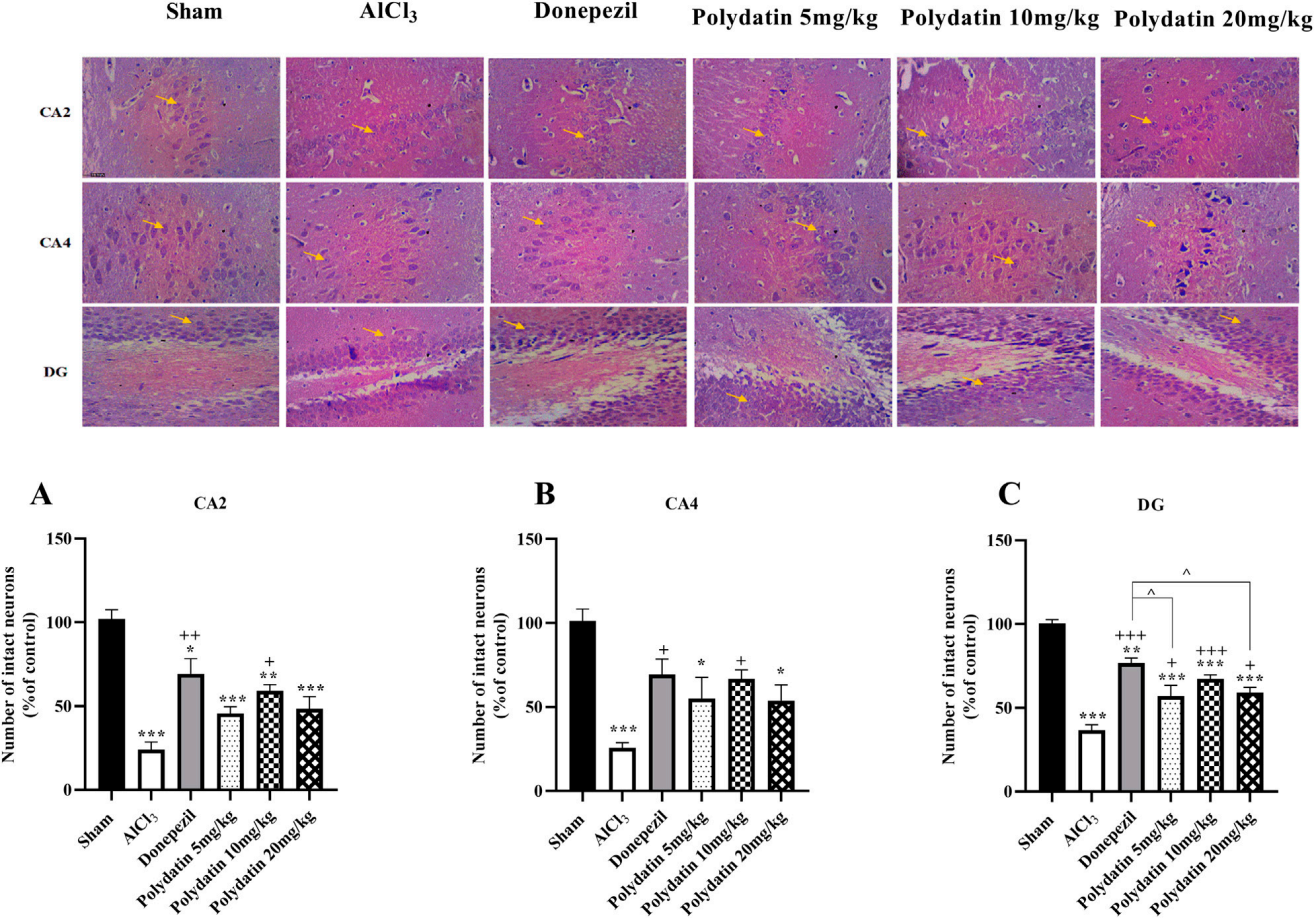
Polydatin impaired histological changes in an AlCl_3_-induced rat model of AD. **(A)** CA2, **(B)** CA4, and **(C)** DG. The data passed the Shapiro-Wilk test for normality. The one-way ANOVA with Tukey *post hoc* analysis was employed, and data were presented as mean ± SEM. *P < 0.05, **P < 0.01, ***P < 0.001 vs. Sham group; ^+^ *P* < 0.05, ^++^ *P* < 0.01, ^+++^ *P* < 0.001 vs. AlCl_3_ group; ^^ *P* < 0.05 vs. Donepezil group.

H&E staining was used to investigate morphological changes in the hippocampus CA2, CA4, and DG regions of experimental rats (Figure 7). The sham group’s neurons showed typical properties such as distinct nuclei, large nucleoli, and abundant cytoplasm, indicating their health. In contrast, treatment with AlCl_3_ resulted in a significant reduction in viable neurons (*P* < 0.001). The group that received 10 or 20 mg/kg of PD showed fewer pathogenic changes in hippocampus neurons compared to the AlCl_3_ group (*P* < 0.05 and *P* < 0.01). There were no significant changes in neuronal health or structure between groups treated with donepezil and those receiving PD at dosages of 10 and 20 mg/kg (*P* < 0.001).

## 4 Discussion

In the present study, we found that rats with AD had reduced rates of learning, recognition memory, and short- and long-term memory. Over 14 days, there was a noticeable drop in motor function along with a decrease in mobility and exploratory behaviors in rats with AD. AD rats showed reduced MMP-2, GSH, and CAT activity, together with increased MMP-9 and nitrite levels. Besides, PD demonstrated anti-inflammatory benefits by increasing the activity of MMP-2, lowering serum nitrite, and enhancing CAT/GSH while inhibiting inflammatory MMP-9. Both behavioral and biochemical indicators showed varied degrees of improvement after PD therapy. Finally, via improving neuronal survival, PD demonstrated neuroprotective benefits in the rat hippocampal CA2, CA4, and DG.

Oxidative stress and inflammation are elevated in the brains of AD patients, and these elevations have been widely confirmed in a variety of model animals. Suppression of the start and/or advancement of AD is a major concern in modern society, and studies have shown that improvements in these pathological circumstances may be advantageous for preserving or enhancing the neuronal functioning of Alzheimer’s patients (Uruno et al., 2020). According to reports, Al is one of the factors that contributes to AD and is deposited in the brain. Important symptoms of Al neurotoxicity, which contributes to the etiology of AD, include Aβ deposition, hyperphosphorylation of tau protein, increased activity of acetylcholinesterase, imbalance in neurotransmitter levels, inflammatory cytokines, mitochondrial damage, oxidative stress, and deficits in memory and learning (Wojtunik-Kulesza et al., 2016; Auti and Kulkarni, 2019). As a result, in this investigation, we employed an animal model of AD caused by AlCl_3_. The current study found that the treatment of AlCl_3_ causes a progressive loss of short-term, spatial, and diagnostic memory in rats. Previous reports have shown the antioxidative potentials of medicinal herbs (Selamoglu-Talas et al., 2015), plant-derived active compounds (e.g., polyphenols and iridoids), and related diets (Vasile et al., 2024) in combating neurodegenerative diseases (Daglia et al., 2014; Sureda et al., 2023; Ishak et al., 2024).

Resveratrol has sparked attention in health studies owing to its therapeutic potential for some neuroprotective properties associated with cognitive impairment. As a polyphenol, resveratrol’s positive benefits have been connected to its action as an antioxidant and anti-inflammatory agent (Yang et al., 2021). Even though the mechanistic insights of resveratrol in AD have been widely examined (Ahmed et al., 2017; Kou and Chen, 2017; Sawda et al., 2017; Drygalski et al., 2018; Gomes et al., 2018), the comparative literature on its role with other stilbenes, particularly PD, is still sparse. PD is a naturally occurring resveratrol glucoside, also known as resveratrol-3-β-mono-D-glucoside derived from the roots of *Reynoutria japonica* Houtt [Polygonaceae]. It is additionally found in grapes, red wines, hop cones, peanuts, cocoa/chocolate goods, and a variety of other foods (Du et al., 2013). It has been observed that PD protects against neurodegenerative disorders such as AD (Tang, 2021), Parkinson’s disease (Huang et al., 2018), cerebral ischemia (Ji et al., 2012; Huang et al., 2018; Shah et al., 2019), traumatic brain injury (Li et al., 2019), and spinal cord injury (Lv et al., 2019b; 2019a).

To examine if PD could have an impact on learning and memory impairment, as well as anxiety-like behaviors, behavioral tests were conducted in AD rats (Webster et al., 2014; Lombardero et al., 2020). The open-field test is used to evaluate the mobility and exploratory behaviors of rats (Prut and Belzung, 2003). Comparing the AlCl_3_ group to the control group, there was a notable drop in the number of crossing lines, rearing, and grooming. Rats receiving PD showed a substantial increase in the number of crossing lines, rearing, and grooming behaviors as compared to the AlCl_3_ group. The passive avoidance test was used to evaluate short-term and long-term memory by recording ITL and STL (Ghafarimoghadam et al., 2022). A noteworthy increase in ITL in rats treated with PD was observed compared to the group treated with AlCl_3_. In this experiment, the increased STL showed improvement in the recognition memory of rats. In addition, the Y-maze test evaluates the spatial memory of the animal based on the percentage of non-repetitive frequency of the total number of entering arms. Healthy animals explore the new arm, whereas animals with peripheral destruction re-enter the old arm that is already been examined. Therefore, compared to healthy animals, a lower number of non-repetitive intervals is registered. The SAP% in the AlCl_3_ group compared to the healthy and treatment groups intensity reduced, indicating a lower rate of learning and development of short-term working memory. This is important because the increased SAP% of rats in the group receiving the PD can be related.

The elevated plus maze test was used to determine learning and memory by measuring the ITL and transfer latency. The reduction in transfer delay time after determining the ITL time indicates the improvement of the rats’ diagnostic memory (Pentkowski et al., 2021). In this test, a significant difference was observed in the learning time between the group receiving AlCl_3_ and the control group. The use of PD improved the ITL (learning) time compared to the AlCl_3_ group.

Oxidative stress acts as a connecting factor between different AD hypotheses and processes. A multifaceted process causes adverse effects on neurons. In AD, oxidative stress is significant and may play a major role in the etiology of the disease (Bai et al., 2022). Because of the crucial roles of oxidative stress in mediating AD difficulties, as well as to obtain further insights into the presumed mechanisms of action of PD, researchers assessed its effects on multiple oxidative stress indicators (GSH, CAT, and NO). This investigation revealed that the group receiving AlCl_3_ had meaningfully higher levels of NO and significantly lower levels of the antioxidant enzymes GSH and CAT as a measure of oxidative stress than the control group. The levels of CAT, GSH, and nitrite were enhanced in this study by PD ingestion.

Neuronal degeneration can be brought on by a variety of mediators released by inflammatory cells. Neuritis may directly cause AD or coexist with other causes of AD to contribute to the disease’s development (Twarowski and Herbet, 2023). Specifically, several neuroinflammatory and neurodegenerative pathways can be triggered by MMP-2 and MMP-9 (Ciccone et al., 2021). Our findings showed that therapy with AlCl_3_ increased inflammation, which eventually led to nerve injury. In this study, administering PD significantly increased MMP-2 levels while reducing MMP-9 levels.

Dementia is linked to the buildup of NFTs in the hippocampus area. The loss of synapses was caused by the buildup of NFT in the stratum lacunosum, dentate fasciculus, CA2, CA3, and CA4 regions. Oxidative damage can impair hippocampal and cortical cognitive function. As an information gateway, the DG plays a major role in the hippocampus’s development of episodic memory (Rao et al., 2022). New evidence indicates that changes in CA4 may be connected with memory processing problems. Integrating information from both the DG and CA4 into larger hippocampal circuits is crucial for efficient memory formation (Aumont et al., 2023). Rats receiving PD at several dosages showed improvements in the histological characteristics of the hippocampus regions CA2, CA4, and DG at therapeutic levels; this was especially true for the group given a high dose of the treatment. Compared with the AlCl_3_ groups, rats receiving PD doses of 10 and 20 mg/kg had the most therapeutic effect, as did the donepezil group.

Despite the effectiveness of resveratrol, an aglycone derivative of PD, it often suffers from low solubility, poor bioavailability, rapid metabolism/clearance, and chemical degradation, thereby limiting associated clinical applications (Tang, 2021). It urges the need for providing PD (as a glycoside resveratrol) and novel delivery formulations. Employing nanotechnology has auspiciously overcome these pharmacokinetic limitations by improving cellular uptake, bioavailability, targeted therapy, and increasing efficacy. Consequently, solid-lipid nanoparticles, liposomes, micelles, and polymeric/metallic nanoparticles have played critical roles in improving the neuroprotective effects of PD. We previously showed the therapeutic effects of PD amphiphilic chitosan nanocarriers against an AlCl_3_-induced rat model of AD (Zarneshan et al., 2025), and the present report confirmed that naïve PD could also play such beneficial effects.

## 5 Conclusion

Using an AlCl_3_-induced AD rat model, the current study examined the therapeutic potential and mechanisms of action of PD against AD. PD dramatically reduced learning and memory impairment as well as anxiety-like behaviors in AD rats, through antioxidant, anti-inflammatory, and neuroprotective mechanisms. Further investigation to establish PD as a novel therapy for AD is needed. To elucidate the more mechanistic pathways and evaluate the long-term safety and efficacy of PD in clinical applications, more studies are required.

## Data Availability

The raw data supporting the conclusions of this article will be made available by the authors, without undue reservation.

## Abbreviations

Aβ, amyloid beta; AD, Alzheimer’s disease; AlCl_3_, aluminum chloride; BBB, blood-brain barrier; CAT, catalase; GSH, glutathione; H&E, Hematoxylin and Eosin; i.p., intraperitoneal; ITL, initial transfer latency time; MMPs, matrix metalloproteinase; NFTs, neurofibrillary tangles; PD, polydatin; STL, step through latency time.
